# Author Correction: Acetylcholine prioritises direct synaptic inputs from entorhinal cortex to CA1 by differential modulation of feedforward inhibitory circuits

**DOI:** 10.1038/s41467-021-27351-z

**Published:** 2021-12-08

**Authors:** Jon Palacios-Filardo, Matt Udakis, Giles A. Brown, Benjamin G. Tehan, Miles S. Congreve, Pradeep J. Nathan, Alastair J. H. Brown, Jack R. Mellor

**Affiliations:** 1 Center for Synaptic Plasticity, School of Physiology, Pharmacology and Neuroscience, University of Bristol, University Walk, Bristol, UK; 2 Sosei Heptares, Steinmetz Building, Granta Park, Great Abingdon, Cambridge, UK; 3 Department of Psychiatry, University of Cambridge, Cambridge, UK; 4 Present Address: OMass Therapeutics Ltd, The Schrödinger Building, Oxford, UK

**Keywords:** Neuroscience, Cellular neuroscience, Neural circuits, Synaptic transmission, Neuroscience, Cellular neuroscience, Neural circuits, Synaptic transmission

Correction to: *Nature Communications* 10.1038/s41467-021-25280-5, published online 16 September 2021.

In this article the grant number 101029/Z/13/Z relating to Wellcome Trust for Jon Palacios-Filardo and Jack R. Mellor and the grant number BB/N013956/1 relating to Biotechnology and Biological Sciences Research Council (BBSRC) for Matt Udakis and Jack R. Mellor were omitted. The original article has been corrected.

