# Supplementary material for: Acetylcholine prioritises direct synaptic inputs from entorhinal cortex to CA1 by differential modulation of feedforward inhibitory circuits
Source: Nat Commun. 2021 Sep 16;12:5475. doi: 10.1038/s41467-021-25280-5 (PMC8445995; doi:10.1038/s41467-021-25280-5)
Supplement: Supplementary file 1 — Supplementary Information [file 41467_2021_25280_MOESM1_ESM.pdf]

Figure S1

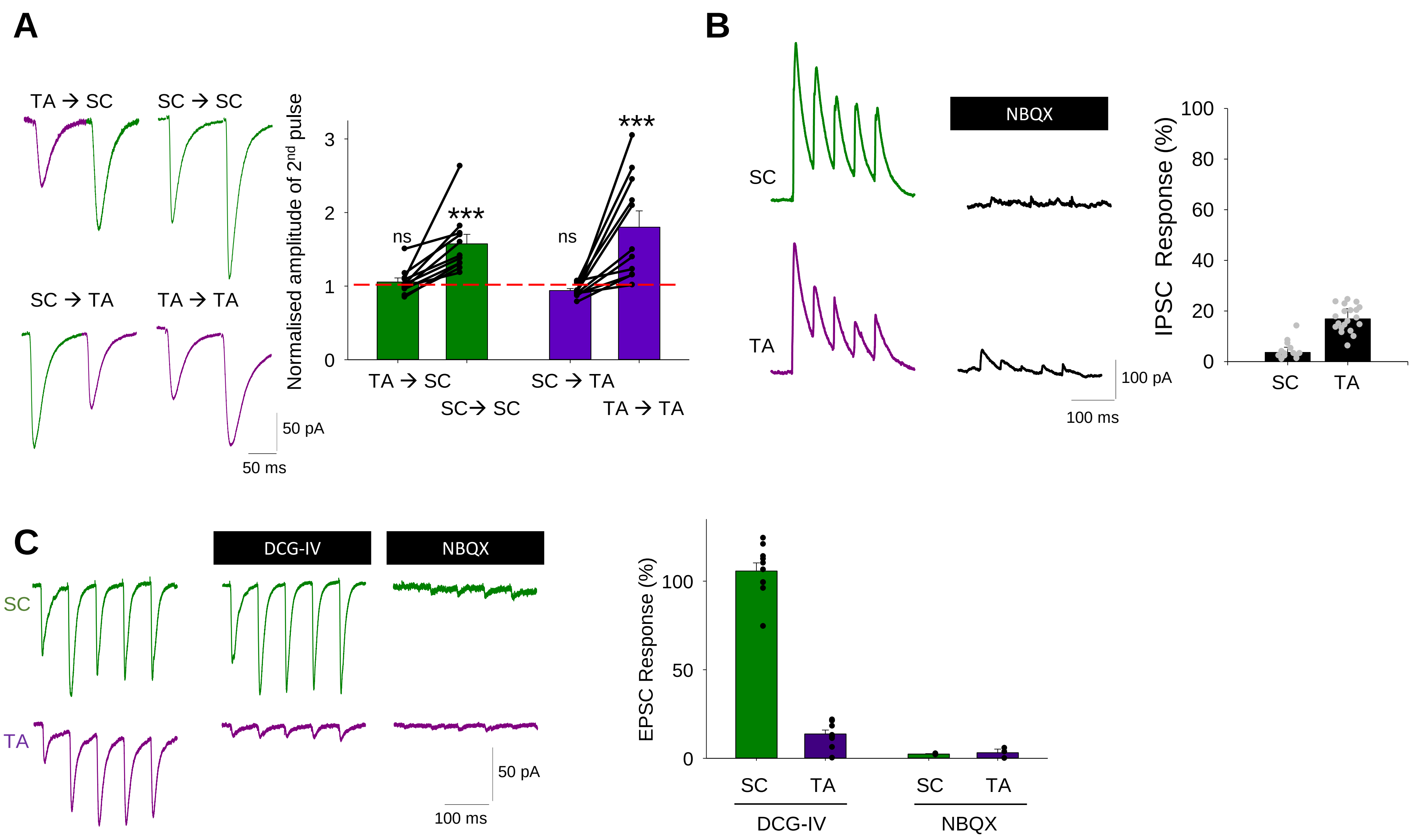

A, Independence of SC (green) and TA (purple) pathways was evaluated by the lack of facilitation of the second EPSC response when the alternate pathway was stimulated (n = 11; TA – SC, p = 0.06; SC – SC, p = 0.0005; SC – TA, p = 0.34; TA – TA, p = 0.0009).

B, Feedforward IPSCs from SC and TA pathways were recorded at 0 mV and confirmed to be disynaptic by sensitivity to NBQX.

C, DCG-IV (3 μM) blocked TA pathway but not SC pathway synaptic responses. Application of AMPA receptor antagonist (NBQX 20 μM) blocked responses in both pathways.

Data are mean ± SEM; Two tailed paired *t*-Test. ***p < 0.001.

Figure S2

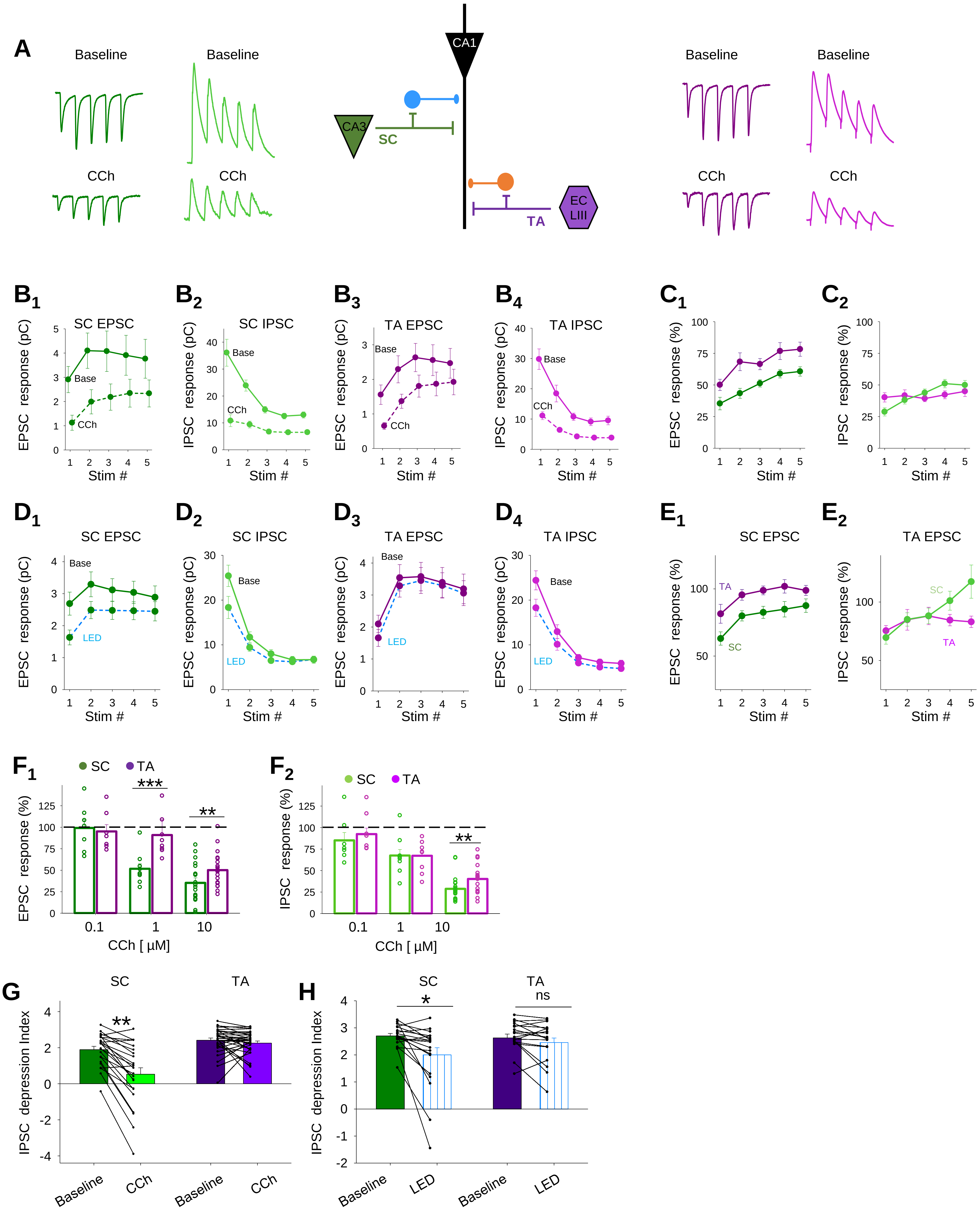

A, Middle, schematic representation of the experimental approach incorporating simultaneous recording of excitatory (V_h = -60 mV) and feedforward inhibitory (V_h = 0 mV) synaptic inputs from Schaffer collateral (SC) and temporoammonic (TA) input pathways to CA1 pyramidal neuron (bottom). Example traces for EPSCs and IPSCs in response to trains of 5 stimuli at 10 Hz to SC (green, left) and TA (purple, right) pathways before and after application of carbachol (CCh, 10 µM).

B, Quantification of SC (B_{1-2}) and TA (B_{3-4}) EPSC ($B_{1,3}$) and IPSC ($B_{2,4}$) charge transfer for each response in the train illustrated in A before and after CCh (10 µM) application.

C, EPSC (C_1) and IPSC (C_2) reduction by CCh for each of the 5 stimuli for SC (green) and TA (purple) pathways shown in A. **D-E**, Same as B and C but for light stimulation of endogenous acetylcholine release relating to Figure 2.

F, Dose-response for CCh depression of EPSCs (F_1) and IPSCs (F_2) for SC (green) and TA (purple) pathways (n = 7; EPSC 1 µM CCh, p = 0.0004; EPSC 10 µM CCh, p = 0.006; IPSC 10 µM CCh, p = 0.002).

G-H, Cholinergic receptor activation by bath applied CCh (G; SC, n = 22, p = 0.009; TA, n = 30, p = 0.821) or light stimulated endogenous acetylcholine release (H; SC, n = 19, p = 0.017; TA, n = 17, p = 0.14) reduced the depression index for SC (green) but not TA (purple) disynaptic feedforward IPSCs. Depression index is calculated as the amount of cumulative depression between the 2nd and 5th responses within the train of 5 responses.

Data are mean ± SEM; Comparisons by two tailed paired *t*-tests *** p < 0.001 ** p < 0.01.

Figure S3

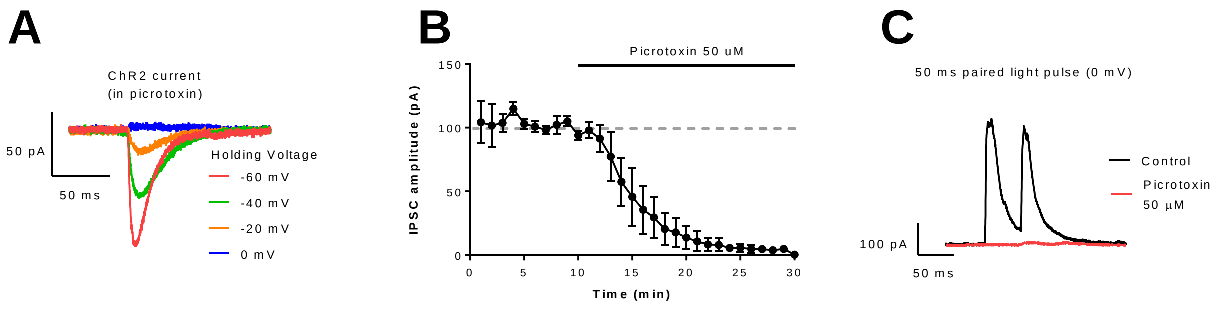

A, ChR2 currents at different holding potentials recorded from a CCK$^+$ and ChR2 expressing pyramidal neuron in response to 2ms light pulses in the presence of picrotoxin (50 μM). At 0mV (the reversal potential for ChR2) no ChR2 currents are observed.

B-C, Light evoked GABAergic responses recorded from pyramidal neurons held at 0mV in the presence of NBQX and DAPV are abolished by picrotoxin (50 μM).

Data are mean ± SEM.

Figure S4

Compound 1	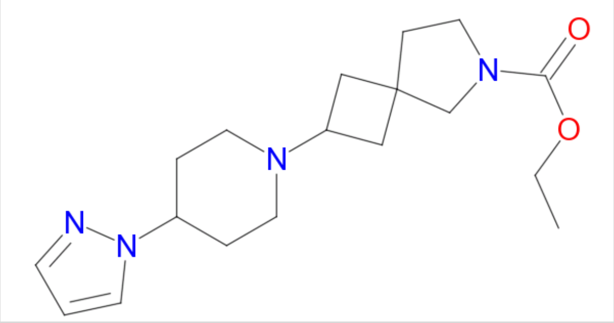	
Parameter	Value[b,c]	N[e]
MWt	332.44	
cLogP / LogD[a]	1.4 / 1.7	
hM_1 pEC50 (Emax)	7.5 ± 0.33 (108)	16
hM_2 pEC50 (Emax)	6.3 ± 0.97 (41)	5
hM_3 pEC50 (Emax)	<4.7	3
hM_4 pEC50 (Emax)	8.4 ± 0.25 (112)	16
rM_4 pEC50 (Emax)	7.6 ± 0.14 (59)	5
hM_2 pKi[d]	6.0 ± 0.23	4

Structure and in vitro pharmacological profile of Compound 1. CHO-K1 cells stably expressing the human M_1–M_4 and rat M_4 receptors were used to determine the pharmacological profile of Compound 1. [a] Calculated LogP value, LogD was measured at pH7.4. [b] Compound pEC50 values were measured using phosphor-ERK format (CisBio). Values reported as <4.7 were considered inactive and did not induce a >10% increase in the response at the highest concentration tested (30µM). [c] The maximum efficacy (Emax values) are expressed as a percentage of the response of a saturating concentration of acetylcholine (1µM) run in the same assay. [d] [3H]-NMS competition binding studies were used to define the affinity (pKi) for Compound 1 at the human muscarinic M_2 receptor. [e] number of replicates. Data are the mean ± S.E.M. Compound 1 can be found within WO2015/118342 which relates to the invention of agonists of the muscarinic M_1 receptor and/or M_4 receptor and which are useful in the treatment of muscarinic M_1/M_4 receptor mediated diseases.

Figure S5

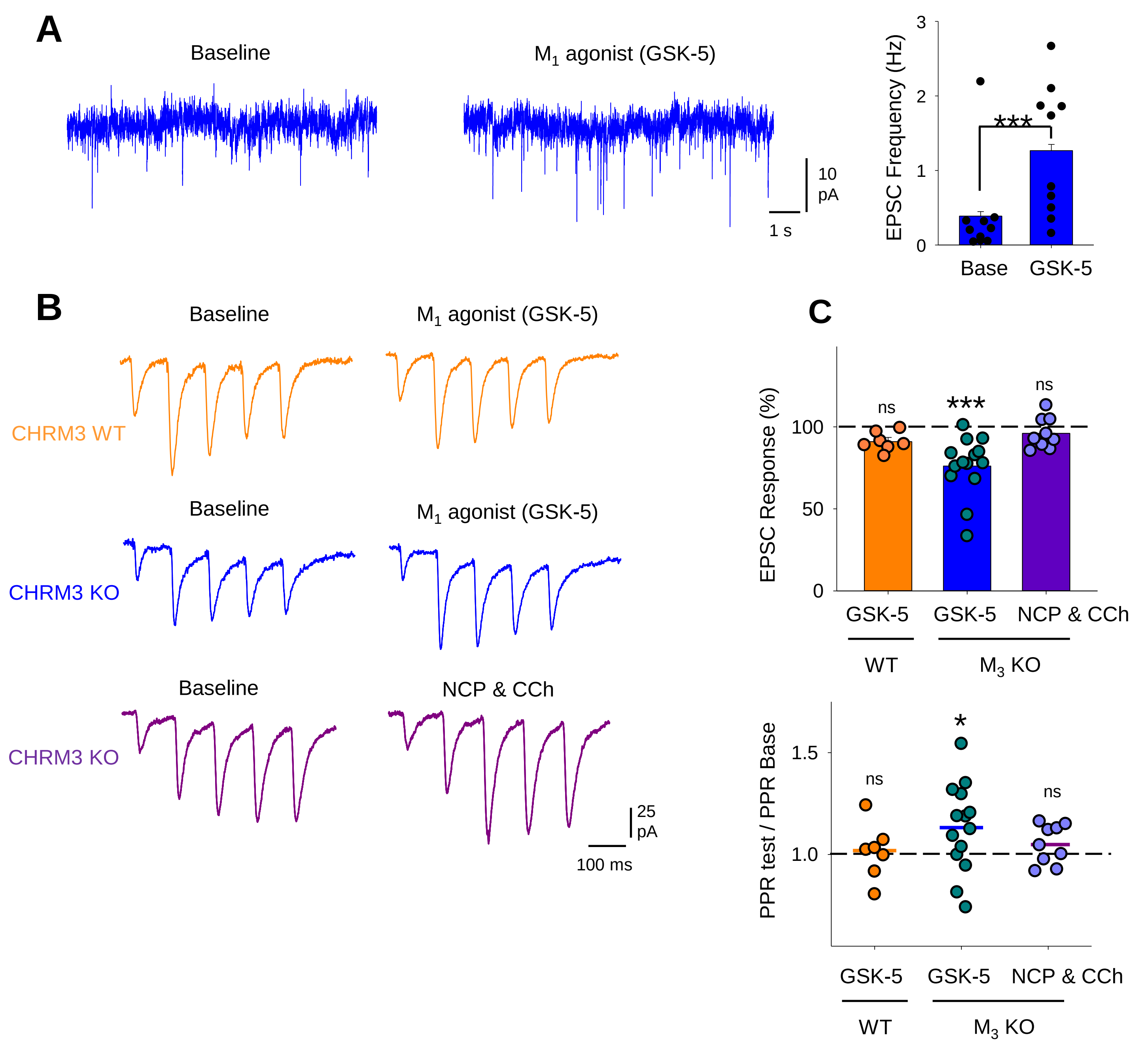

A, Muscarinic M_1 receptor agonist (GSK-5, 500nM) produced an increase in the frequency of spontaneous excitatory events recorded from CA1 pyramidal neurons (n = 10, p = 0.0008).

B-C, GSK-5 caused a reduction of TA pathway EPSC (B) and an increase of PPR (C) in slices from CHRM3 KO mice but not in slices from CHRM3 WT mice. Nitrocaramiphen (1 µM) prevented the reduction in TA pathway EPSC (B) and increase in PPR (C) caused by carbachol in slices from CHRM3 KO mice (WT GSK-5 EPSC, n = 7, p = 0.122; WT GSK-5 PPR, p = 0.81; M3 KO GSK-5 EPSC, n = 14, p = 0.0001; M3 KO GSK-5 PPR, p = 0.039; M3 KO CCh + NCP EPSC, n = 9, p = 0.242; M3 KO CCh + NCP PPR, p = 0.168).

Data are mean ± SEM; Comparisons by two tailed paired *t*-tests *** p < 0.001 * p < 0.05.

Figure S6

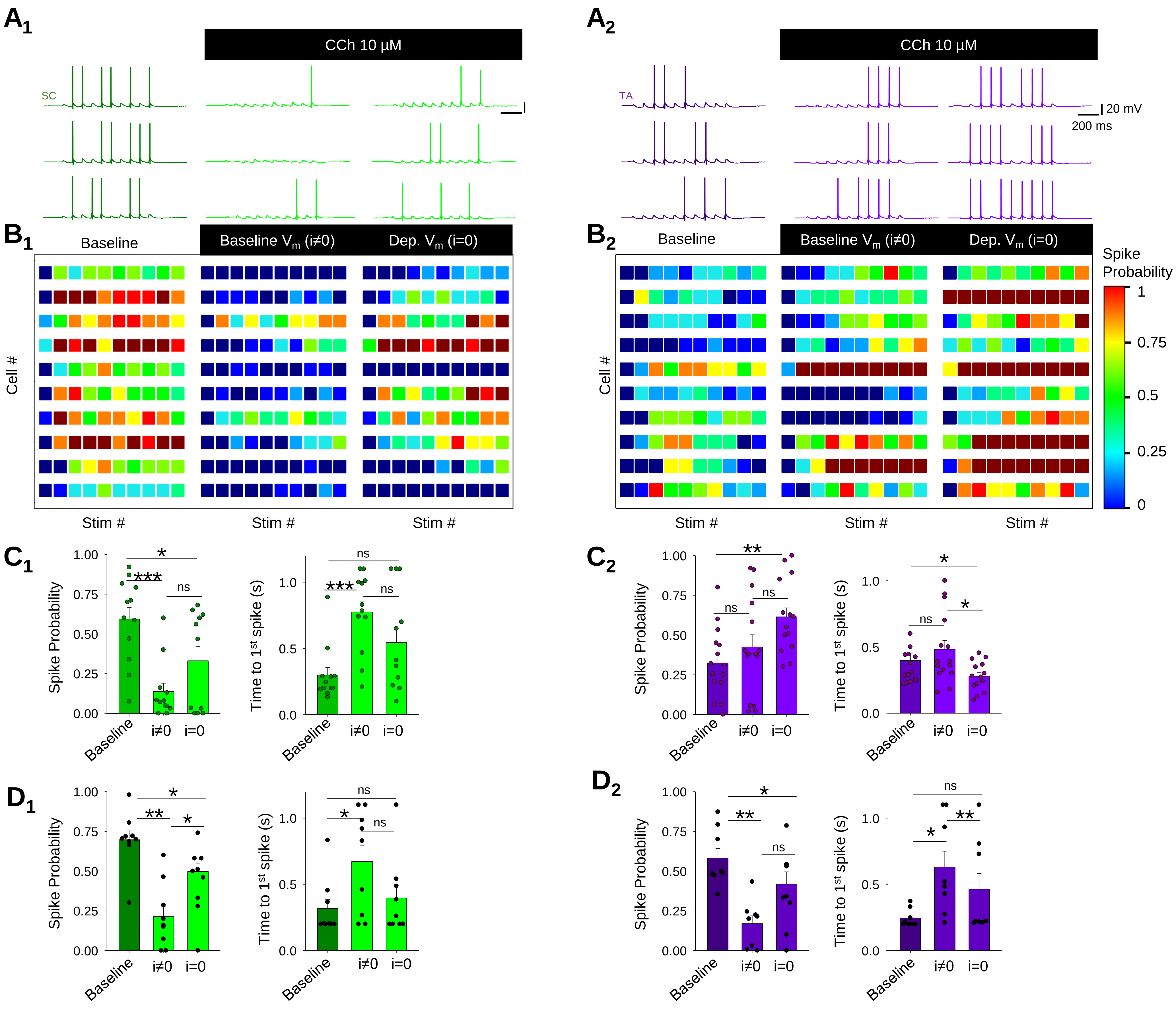

A, Responses in CA1 pyramidal neurons to 10 stimuli at 10 Hz given to Schaffer collateral (SC, A_1) or temporoammonic (TA, A_2) input pathways. After application of CCh (10 µM), membrane potential (V_h) is initially held at baseline levels by injection of current (i≠0) and then allowed to depolarise (i=0).

B, Heat maps depicting spike probability for 10 stimulation pulses from 10 cells for SC (B_1) and TA (B_2) input pathways before and during CCh application.

C, Spike probability and time to first spike for SC (C_1; n = 20 from 11 mice; spike probability I ≠ 0, $p < 0.0001$; spike probability I = 0, p = 0.024; I ≠ 0 vs I = 0, p = 0.052; time to first spike I ≠ 0, $p < 0.0001$; time to first spike I = 0, p = 0.077; I ≠ 0 vs I = 0, p = 0.087) and TA (C_2; n = 20 from 11 mice; spike probability I ≠ 0, p = 0.162; spike probability I = 0, p = 0.002; I ≠ 0 vs I = 0, p = 0.064; time to first spike I ≠ 0, p = 0.087; time to first spike I = 0, p = 0.031; I ≠ 0 vs I = 0, p = 0.017) input pathways. Spike probability decreased after CCh application in SC pathway but increased in TA pathway.

D, In the presence of $GABA_A$ receptor antagonist, CCh reduced spike probability and increased time to spike in both SC (D_1; n = 20 from 11 mice; spike probability I ≠ 0, p = 0.003; spike probability I = 0, p = 0.024; I ≠ 0 vs I = 0, p = 0.032; time to first spike I ≠ 0, p = 0.023; time to first spike I = 0, p = 0.077; I ≠ 0 vs I = 0, p = 0.067) and TA (D_2; n = 20 from 11 mice; spike probability I ≠ 0, p = 0.004; spike probability I = 0, p = 0.024; I ≠ 0 vs I = 0, p = 0.162; time to first spike I ≠ 0, p = 0.011; time to first spike I = 0, p = 0.091; I ≠ 0 vs I = 0, p = 0.007) input pathways.

Data are mean ± SEM; Comparisons by one-way ANOVA with repeated measures and post hoc Bonferroni correction *** $p < 0.001$ ***$p < 0.01$ * $p < 0.05$.

Figure S7

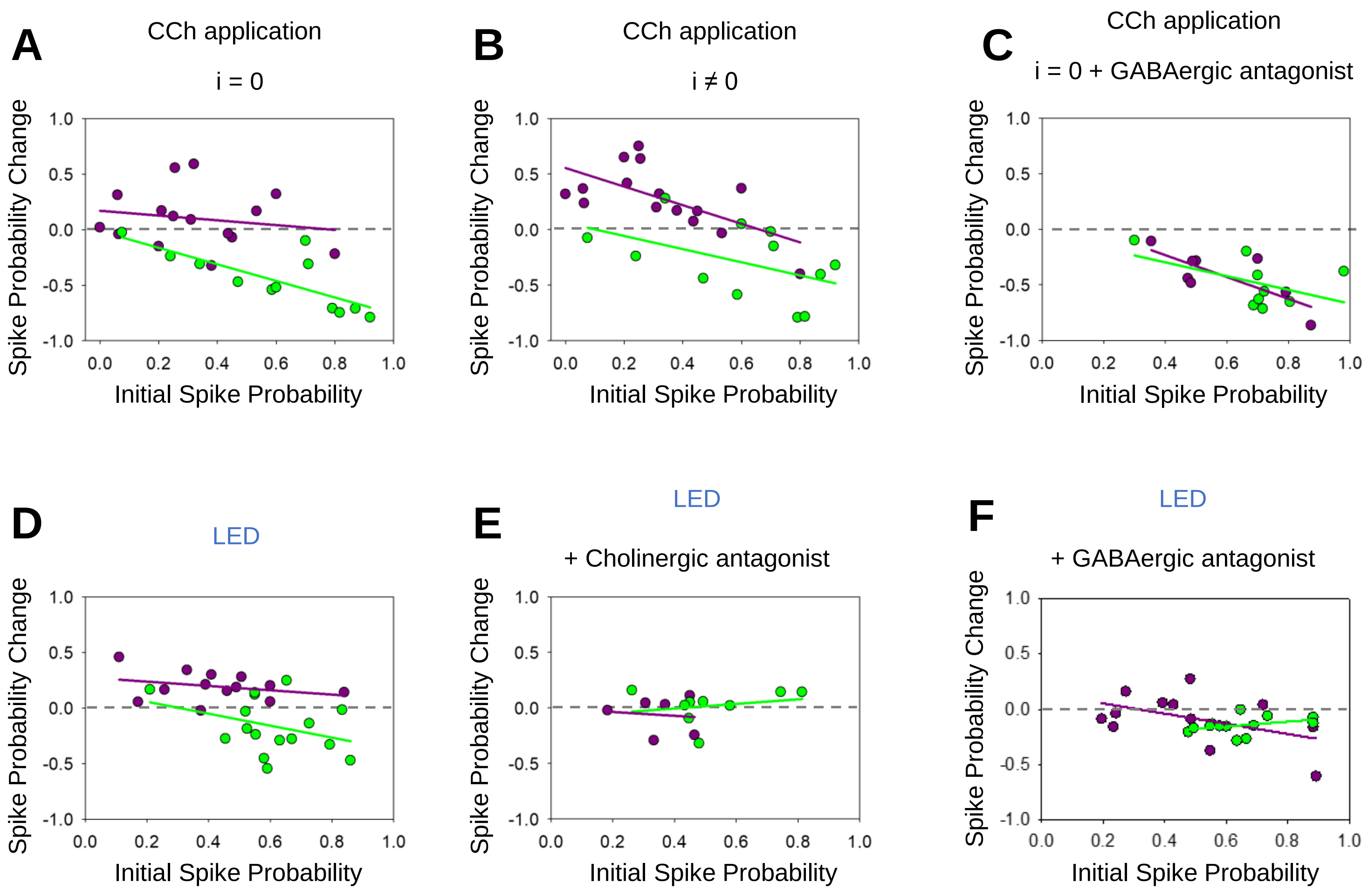

A-F, Spike probability changes correlated with the initial spike probability for each pathway (SC green and TA purple) in each experimental condition (A-C, related to Figure S6; D-E, related to Figure 6; F, related to Figure 7). Linear regression trend is shown as solid line for each pathway.

Figure S8

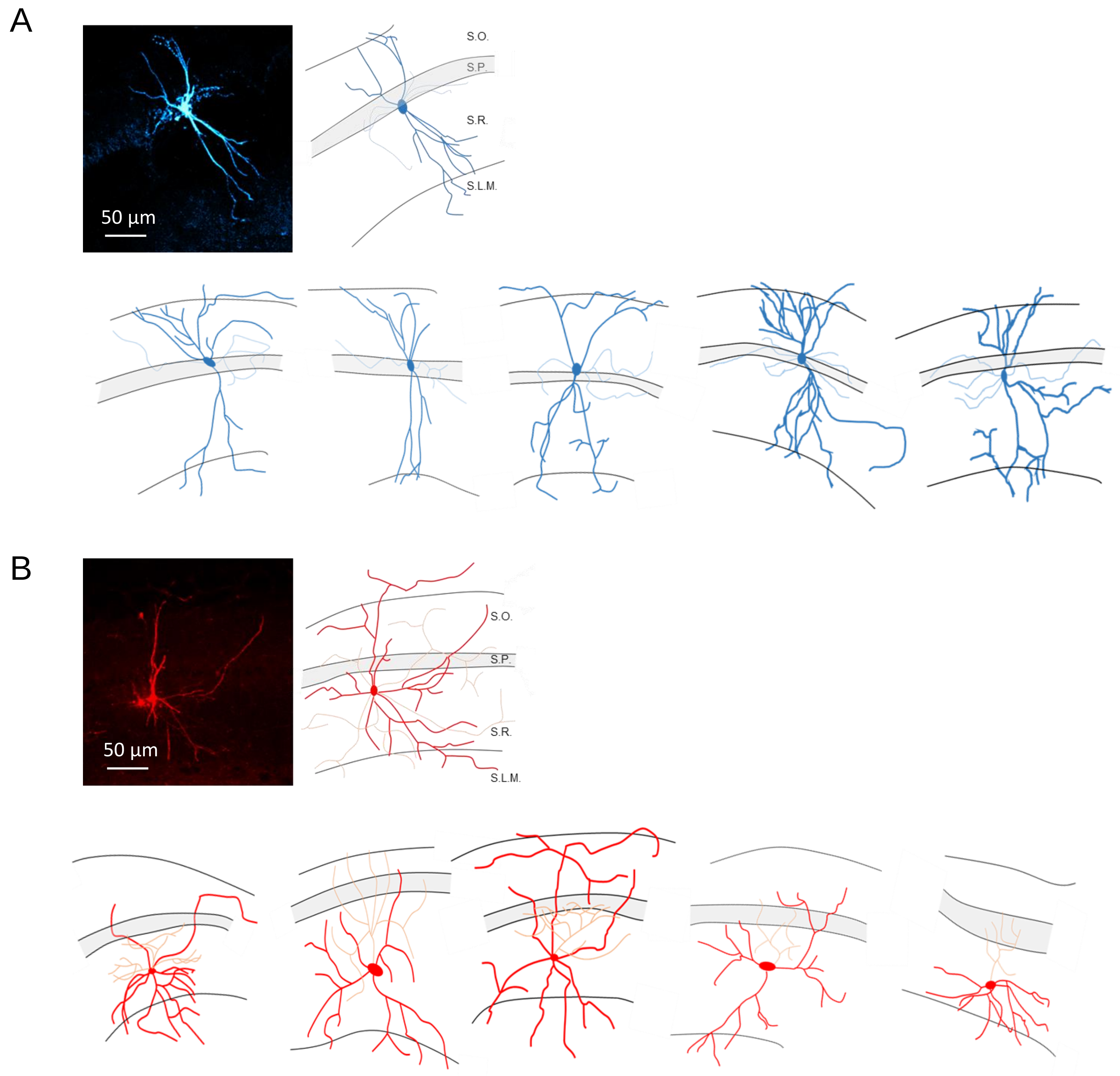

A-B, Images of 6 neurolucida-traced PV (A) or CCK (B) expressing interneurons filled with neurobiotin and co-immunostained for PV (A) or CCK (B) to confirm neurochemical phenotype. Dendrites are in bold and axons in faint lines. Example neurobiotin staining shown top left for each interneuron type.
