# Supplementary material for: Acetylcholine prioritises direct synaptic inputs from entorhinal cortex to CA1 by differential modulation of feedforward inhibitory circuits
Source: Nat Commun. 2021 Sep 16;12:5475. doi: 10.1038/s41467-021-25280-5 (PMC8445995; doi:10.1038/s41467-021-25280-5)
Supplement: Supplementary file 3 — Reporting Summary [file 41467_2021_25280_MOESM3_ESM.pdf]

nature research

Corresponding author(s): Jack Mellor

Last updated by author(s): Jun 18, 2021

Reporting Summary

Nature Research wishes to improve the reproducibility of the work that we publish. This form provides structure for consistency and transparency in reporting. For further information on Nature Research policies, see Authors & Referees and the Editorial Policy Checklist .

Statistics

For all statistical analyses, confirm that the following items are present in the figure legend, table legend, main text, or Methods section.

n/a	Confirmed	
[ ]	[x]	The exact sample size (*n*) for each experimental group/condition, given as a discrete number and unit of measurement
[ ]	[x]	A statement on whether measurements were taken from distinct samples or whether the same sample was measured repeatedly
[ ]	[x]	The statistical test(s) used AND whether they are one- or two-sided<br>*Only common tests should be described solely by name; describe more complex techniques in the Methods section.*
[x]	[ ]	A description of all covariates tested
[x]	[ ]	A description of any assumptions or corrections, such as tests of normality and adjustment for multiple comparisons
[ ]	[x]	A full description of the statistical parameters including central tendency (e.g. means) or other basic estimates (e.g. regression coefficient) AND variation (e.g. standard deviation) or associated estimates of uncertainty (e.g. confidence intervals)
[ ]	[x]	For null hypothesis testing, the test statistic (e.g. *F*, *t*, *r*) with confidence intervals, effect sizes, degrees of freedom and *P* value noted<br>*Give P values as exact values whenever suitable.*
[x]	[ ]	For Bayesian analysis, information on the choice of priors and Markov chain Monte Carlo settings
[x]	[ ]	For hierarchical and complex designs, identification of the appropriate level for tests and full reporting of outcomes
[x]	[ ]	Estimates of effect sizes (e.g. Cohen's *d*, Pearson's *r*), indicating how they were calculated

Our web collection on statistics for biologists contains articles on many of the points above.

Software and code

Policy information about availability of computer code

Data collection	Electrophysiology data was acquired via Signal software (CED)
Data analysis	Electrophysiology data was analysed via Signal (CED), Graphpad (Prism) and Matlab

For manuscripts utilizing custom algorithms or software that are central to the research but not yet described in published literature, software must be made available to editors/reviewers. We strongly encourage code deposition in a community repository (e.g. GitHub). See the Nature Research guidelines for submitting code & software for further information.

Data

Policy information about availability of data

All manuscripts must include a data availability statement. This statement should provide the following information, where applicable:

- Accession codes, unique identifiers, or web links for publicly available datasets
- A list of figures that have associated raw data
- A description of any restrictions on data availability

Further information and data that support the findings of this study are available from the corresponding author Jack.mellor@bristol.ac.uk or upon reasonable request.

Field-specific reporting

Please select the one below that is the best fit for your research. If you are not sure, read the appropriate sections before making your selection.

[x] Life sciences [ ] Behavioural & social sciences [ ] Ecological, evolutionary & environmental sciences

Life sciences study design

All studies must disclose on these points even when the disclosure is negative.

Sample size	Sample size was calculated as n=6 via power calculations using expected effect size and variability based on previous experiments, with power set at 80% and alpha set at 0.05
Data exclusions	For synaptic amplitude experiments data were excluded if the control pathway deviated >50% or the Series resistance deviated >20% from baseline values.
Replication	For electrophysiology experiments the experimental unit was defined as cell with only one cell recorded per slice. Up to 3 cells were recorded from each animal with an average of 1.6 cells per animal.
Randomization	pharmacological experiments were conduced in parallel with control experiments and where possible the experimental condition was randomly assigned.
Blinding	Blinding was not generally possible except for data using M3 KO mice where the experimenter was blinded to genotype, however experiments were conducted with a within cell control used as an exclusion criteria.

Reporting for specific materials, systems and methods

We require information from authors about some types of materials, experimental systems and methods used in many studies. Here, indicate whether each material, system or method listed is relevant to your study. If you are not sure if a list item applies to your research, read the appropriate section before selecting a response.

Materials & experimental systems

n/a	Involved in the study	
☐	☒	Antibodies
☒	☐	Eukaryotic cell lines
☒	☐	Palaeontology
☐	☒	Animals and other organisms
☒	☐	Human research participants
☒	☐	Clinical data

Methods

n/a	Involved in the study	
☒	☐	ChIP-seq
☒	☐	Flow cytometry
☒	☐	MRI-based neuroimaging

Antibodies

Antibodies used	Alexa- 594 anti-mouse (raised in goat) FisherThermo CatNO. A11032
Validation	Antibodies have been used in multiple peer reviewed articles that can be found alongside validation on the manufacturers website.

Animals and other organisms

Policy information about studies involving animals; ARRIVE guidelines recommended for reporting animal research

Laboratory animals	several genetically modified mice were used:<br>Ai32 mice (Gt(ROSA)26Sortm32(CAG-COP4*H134R/EYFP)Hze Jax Stock number: 024109)<br>PV-Cre (Pvalbtm1(cre)Arbr/J Jax stock number: 017320)<br>CCK-Cre (Ccktm1.1(cre)Zjh/J Jax stock number: 012706)<br>ChaT-cre (Chattm2(cre)Lowl Jax stock number: 006410)<br>CHRM3 knockout mice (Chrm3tm1Jwe/J Jax stock number: 030163)<br><br>All mice were on a C57BL/6J background and both male and female mice were used between ages of 4-9 weeks.
Wild animals	study did not involve wild animals
Field-collected samples	study did not involve samples collected from the field
Ethics oversight	All procedures and techniques were conducted in accordance to the UK animals scientific procedures act, 1986 with approval of the University of Bristol ethics committee.

Note that full information on the approval of the study protocol must also be provided in the manuscript.
